# Francisella tularensis Exploits AMPK Activation to Harvest Host-Derived Nutrients Liberated from Host Lipolysis

**DOI:** 10.1128/iai.00155-22

**Published:** 2022-08-02

**Authors:** Sedelia R. Dominguez, Shannon Whiles, Kelly N. Deobald, Thomas Kawula

**Affiliations:** a Paul G. Allen School for Global Health, Washington State University, Pullman, Washington, USA; b School of Molecular Biosciences, Washington State University, Pullman, Washington, USA; Stanford University

**Keywords:** *Francisella tularensis*, carbon metabolism, host-pathogen interactions, infectious disease, nutritional immunity, zoonotic infections

## Abstract

Francisella tularensis is a zoonotic, facultative intracellular bacterial pathogen that replicates in a variety of cell types during infection. Following entry into the cell and phagosome escape, the bacterium replicates rapidly in the cytoplasm. F. tularensis intracellular growth depends on the availability of metabolizable essential nutrients to support replication. However, the mechanism by which metabolizable nutrients become available to the bacterium in the intracellular environment is not fully understood. We found that F. tularensis-infected cells had significantly smaller and fewer lipid droplets than uninfected cells. Inhibition of triacylglycerol degradation significantly reduced bacterial growth, whereas inhibition of triacylglycerol formation did not reduce bacterial growth, suggesting that triacylglycerols sequestered within lipid droplets are important nutrient sources for F. tularensis. We found that F. tularensis-infected cells had increased activation of lipolysis and the upstream regulatory protein AMP protein kinase (AMPK). These data suggest that F. tularensis exploits AMPK activation and lipid metabolism to use host-derived nutrients. Finally, we found that AMPK activation is correlated with an increased bacterial burden, which suggests that it is a host-mediated response to nutrient starvation that results from increased bacterial replication. Altogether, we conclude that F. tularensis exploits AMPK activation to access nutrients sequestered in lipid droplets, specifically glycerol and fatty acids, to undergo efficient bacterial replication and cause successful infection.

## INTRODUCTION

Access to metabolizable nutrients within an infected host is a critical component of pathogen replication and survival. Although nutrient sources are abundant in host cells, most nutrients are stored or sequestered within complex structures such as proteins or lipid droplets (1–3). Therefore, pathogens must possess strategies to liberate nutrients from these nutrient-rich sources within the cell or compete with host metabolic processes to promote replication within their specialized niche while avoiding cellular immune defenses (2–4). Currently, much remains to be learned about how pathogens access key nutrient sources to support replication and cause successful infection.

Francisella tularensis is a Gram-negative, facultative intracellular bacterial pathogen that infects a wide range of species (5). Following entry into the cell, the bacterium initially resides within a phagosome, which is rapidly degraded (6). Upon release into the cytoplasm, the bacterium undergoes replication, leading to host cell death, bacterial release, and subsequent infection of neighboring cells (6–8). Additionally, the bacterium can infect neighboring cells by transferring between two mononuclear cells through a process termed merocytophagy (9, 10). During infection, F. tularensis replication depends on the use and availability of various cellular nutrients (11–14). For example, we and others have demonstrated that F. tularensis assimilates nonglucose carbon sources and that glucose is dispensable for sufficient bacterial replication *in vitro* and virulence in an animal model (12, 15). Instead, F. tularensis replication requires the use of gluconeogenesis, and gluconeogenic products (i.e., glycerol and glycerol-3-phosphate) are important carbon sources for the bacterium (12, 15–17). Therefore, in this study, we aimed to define how these nutrient sources become available in F. tularensis-infected cells.

AMP protein kinase (AMPK) is a cellular protein involved in regulating metabolic pathways according to cellular energy demands (18–21). In mammals, AMPK is activated through phosphorylation at residue Thr172 in response to an increased AMP-to-ATP ratio (18–21). As a result, activated AMPK switches off anabolic pathways and switches on catabolic pathways (18–21). For example, AMPK activation will activate or inactivate enzymes that control nutrient production and regulate the expression of genes involved in cellular metabolism (20, 22, 23). One important area of AMPK regulation is lipid metabolism. The activation of AMPK will inhibit lipid synthesis by targeting acetyl-coenzyme A (CoA) carboxylase (ACC), which is an enzyme that converts acetyl-CoA to malonyl-CoA in the cell (24). Malonyl-CoA is a suppressor of the fatty acid transport protein carnitine palmitoyl transferase 1 (CPT1). When AMPK is active, ACC will be phosphorylated, which prevents this conversion step and therefore allows fatty acids to be transported into the mitochondria for fatty acid oxidation (or β-oxidation) (22, 25, 26). Additionally, AMPK activation can lead to the activation of adipose triglyceride lipase (ATGL) and hormone-sensitive lipase (HSL), two enzymes involved in the degradation of triacylglycerol within lipid droplets (also known as lipolysis) (22, 25). As a result, the activation of ATGL promotes the release of fatty acids and glycerol sequestered in lipid droplets to be translocated to mitochondria for β-oxidation. Therefore, the activation of AMPK will upregulate lipid metabolism in the cell.

It is well established that the activation or inhibition of AMPK or AMPK-regulated processes play important roles in viral survival and replication (22). However, the contribution of AMPK and its downstream processes is not fully understood for bacterial infections. Therefore, to identify the mechanism by which host-derived gluconeogenic carbon sources become available in F. tularensis-infected cells, we aimed to investigate the relationship between AMPK activation and host lipid metabolism during F. tularensis infection.

## RESULTS

### Lipid droplets are degraded during F. tularensis infection.

**FIG 1:**
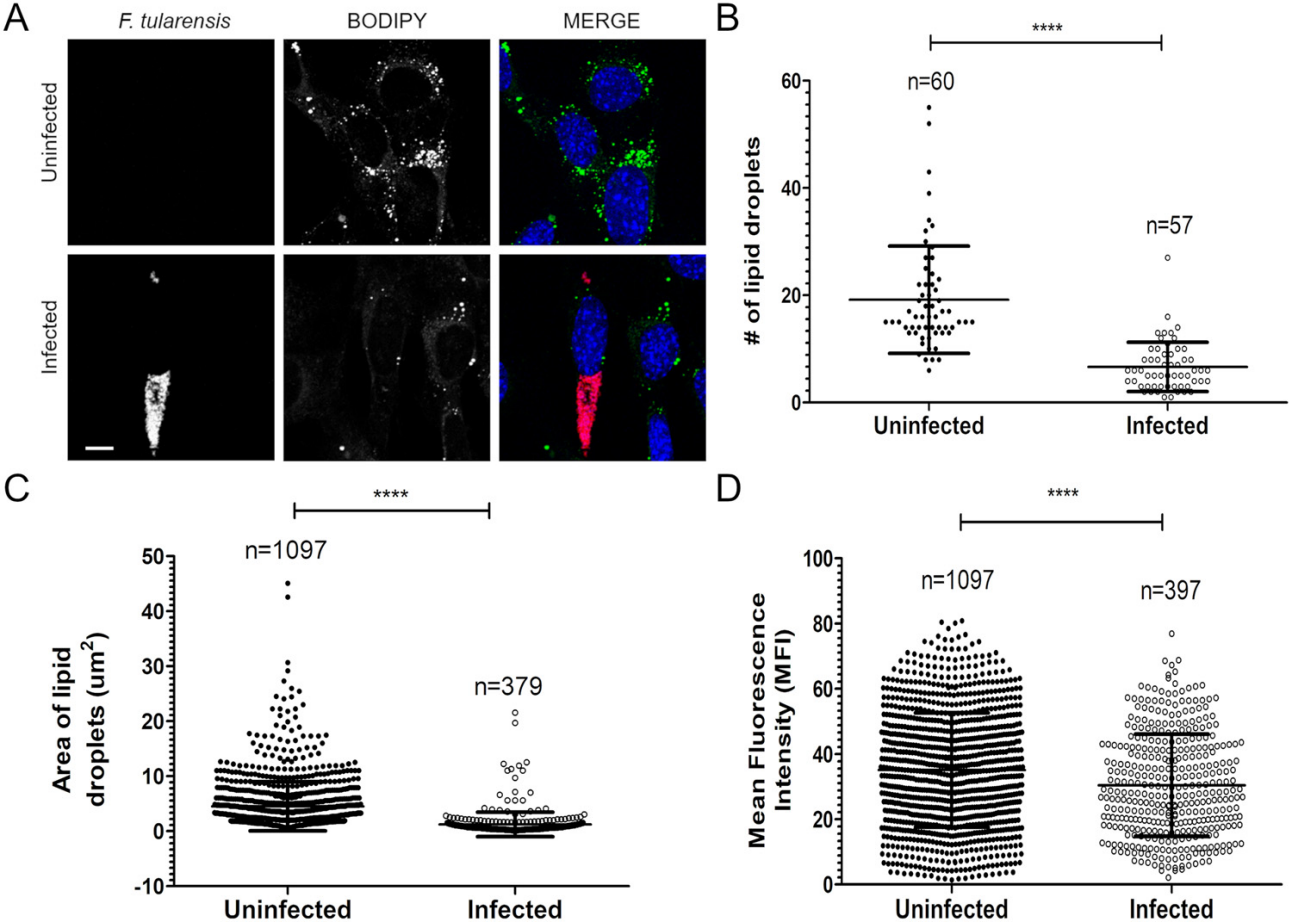
Lipid droplets are degraded in F. tularensis-infected cells. (A) Representative confocal fluorescence micrographs of WT MEFs infected with WT F. tularensis for 36 h. Cells were stained with BODIPY 493/503 (green) and anti-F. tularensis LPS conjugated to Alexa Fluor 647 (red). Bar, 5 μm. (B) Quantification of total lipid droplets per cell as determined by ImageJ. Data represent the means ± standard deviations (SD) for ~60 cells from >3 independent biological replicates. Asterisks represent significant differences between the total numbers of lipid droplets as determined by Student’s unpaired *t* test. ****, *P* < 0.0001. (C) Quantification of the lipid droplet area (square micrometers) as determined by ImageJ. Data represent the means ± SD for total lipid droplets quantified in panel B in cells from >3 independent biological replicates. Asterisks represent significant differences as determined by Student’s unpaired *t* test. ****, *P* < 0.0001. (D) Mean fluorescence intensity of lipid droplets as determined by ImageJ. Data represent the means ± SD for total lipid droplets quantified in panel B in cells from >3 independent biological replicates. Asterisks represent significant differences as determined by Student’s unpaired *t* test. ****, *P* < 0.0001.

We have previously shown that F. tularensis exploits alternate carbon metabolism pathways depending on the availability of host-derived carbon sources (12, 27). F. tularensis requires the use of gluconeogenic carbon sources, some of which are sequestered in lipid droplets in cells (28). To understand how F. tularensis accesses gluconeogenic carbon sources during infection, we investigated the role of lipid droplet degradation during infection. F. tularensis invades and replicates in many different cell types (11, 12). F. tularensis can replicate and survive in fibroblasts. In fact, F. tularensis growth in fibroblasts is prolonged, with peak infection at 36 h (11). Additionally, fibroblasts are a cell line that has an abundance of lipid droplets that can be clearly quantified. Therefore, we used mouse embryonic fibroblasts (MEFs) to investigate the importance of lipid droplet metabolism during F. tularensis infection. We infected MEFs with wild-type (WT) F. tularensis Schu S4 and analyzed the number, area, and mean fluorescence intensity (MFI) of 4,4-Difluoro-1,3,5,7,8-Pentamethyl-4-Bora-3a,4a-Diaza-s-Indacene (BODIPY)-stained lipid droplets at 36 h postinoculation (p.i.). At earlier time points or in cells in which the bacterial burden was not completely established, we did not observe significant differences in lipid droplet staining (data not shown). However, at 36 h p.i., we found that infected MEFs had significantly fewer lipid droplets/cell than did uninfected cells (Fig. 1A). Specifically, infected MEFs had between 5 and 8 lipid droplets/cell, compared to 17 to 22 lipid droplets/cell in uninfected MEFs (Fig. 1B). Furthermore, the area of lipid droplets in uninfected MEFs was 4.5 μm^2^ ± 0.3 μm^2^, which was significantly larger than area of lipid droplets in infected cells (Fig. 1C). In agreement with this conclusion, the MFI of lipid droplets decreased significantly in F. tularensis-infected cells compared to uninfected cells (Fig. 1D). These results suggest that F. tularensis-infected cells have less lipid droplet accumulation than uninfected cells.

### Targeting lipid droplet metabolism alters F. tularensis intracellular growth.

**FIG 2:**
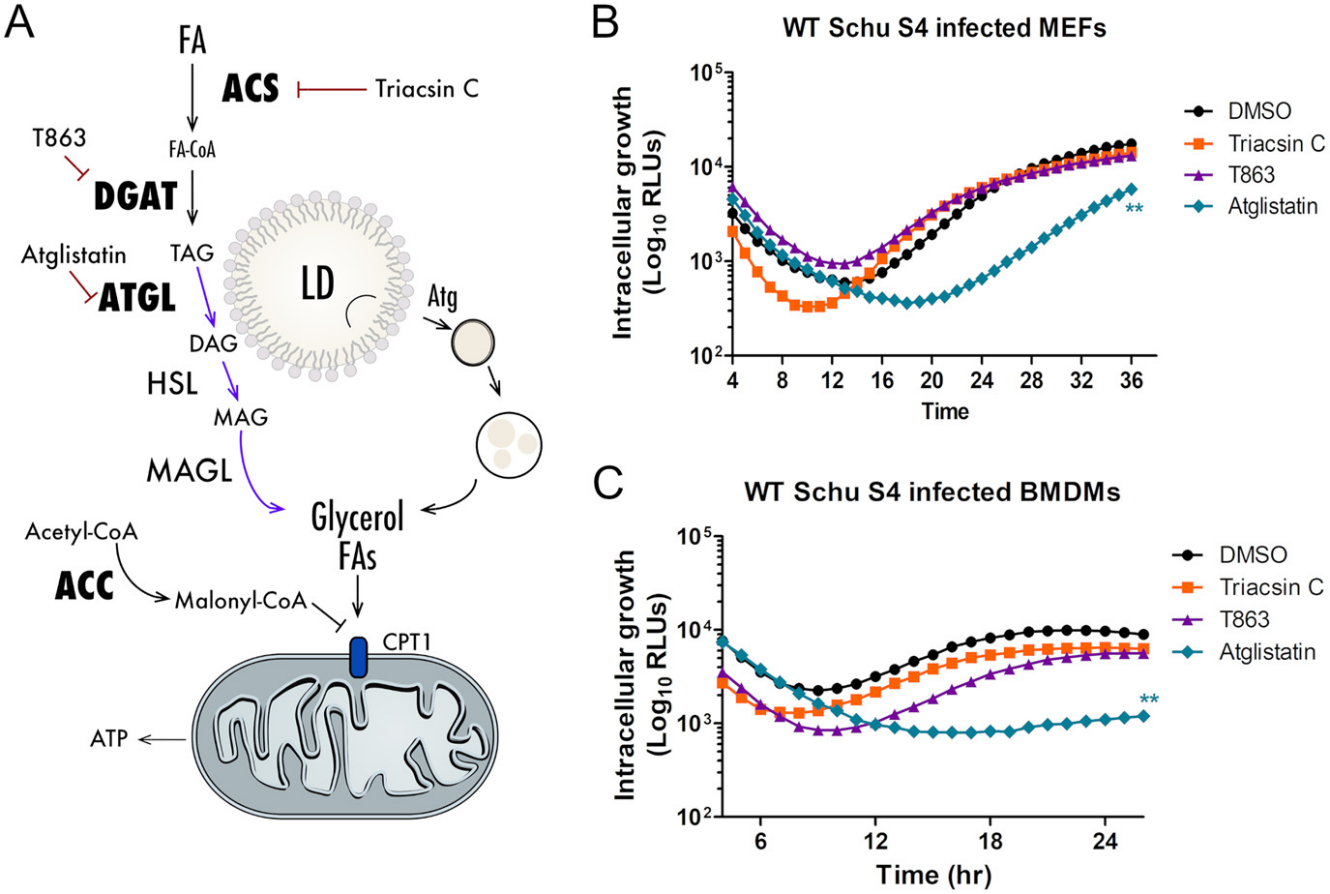
Targeting lipid droplet homeostasis has various effects on F. tularensis intracellular growth. (A) Lipid droplet (LD) homeostasis pathways. ACS, acyl CoA synthetase; DGAT, diacylglycerol acyltransferase; ATGL, adipose triglyceride lipase; ACC, acetyl-CoA carboxylase; FA, fatty acid. TAG, triacylglycerol; DAG, diacylglycerol; MAG, monoacylglycerol; MAGL, monoacylglycerol lipase. (B) Growth kinetics of WT MEFs treated with 30 μM atglistatin, 5 μM triacsin C, 10 μM T863, or DMSO as a control and infected with WT F. tularensis Schu S4 harboring a luciferase plasmid (LUX). Luminescence (RLU) was measured over 24 h. Data points are the means ± SD and represent results from 3 biological replicates performed in triplicate. (C) Growth kinetics of BMDMs treated with 30 μM atglistatin, 5 μM triacsin C, 10 μM T863, or DMSO as a control and infected with WT F. tularensis Schu S4 harboring a luciferase plasmid (LUX). Luminescence (RLU) was measured over 24 h. Data points are the means ± SD and represent the results of 3 biological replicates performed in triplicate.

Given our findings that F. tularensis-infected cells have decreased lipid droplets, we further assessed the importance of lipid droplet homeostasis during F. tularensis infection. We reasoned that targeting lipid droplet formation and degradation during infection would impact F. tularensis replication. We infected cells with F. tularensis harboring a luminescence reporter plasmid (luciferase [LUX]) to quantitate the growth kinetics in WT MEFs. We have previously shown that measuring intracellular growth via luminescence (relative luminescence units [RLU]) is proportional to growth measured via dilution plating (CFU) (29). We treated MEFs with three different inhibitors, triacsin C, T863, and atglistatin. Triacsin C inhibits acyl coenzyme A (CoA) synthetase (ACS), which mediates one of the first steps for fatty acids to be stored in lipid droplets (Fig. 2A). T863 inhibits diacylglycerol acyltransferase 1 (DGAT1), which is responsible for the final step in triacylglycerol formation prior to storage in lipid droplets (Fig. 2A). Finally, we treated cells with atglistatin, which inhibits adipose triglyceride lipase (ATGL), an enzyme responsible for the breakdown of triacylglycerols from lipid droplets (Fig. 2A) (30, 31). We confirmed the activity of these compounds by quantifying the number of lipid droplets following treatment overnight via immunofluorescence microscopy (Fig. S1). Interestingly, we found that bacterial replication was not altered upon treatment with triacsin C or T863 (Fig. 2B). In contrast, treatment with atglistatin, which we have previously shown to inhibit the replication of F. tularensis in macrophages, resulted in a significant reduction in bacterial growth in MEFs (Fig. 2B) (12). Importantly, atglistatin did not directly inhibit F. tularensis growth in broth at various doses (Fig. S1). Similar growth kinetics were observed in bone marrow-derived macrophages (BMDMs), a cell type that F. tularensis primarily infects (Fig. 2C). These results suggest that the accumulation of lipid droplets negatively impacts F. tularensis bacterial growth. Altogether, these results support the conclusion that F. tularensis exploits lipid droplet homeostasis to use sequestered nutrients, which supports our previous findings that F. tularensis-infected cells have decreased lipid droplets (Fig. 1).

### Lipolysis is activated in F. tularensis-infected cells.

**FIG 3:**
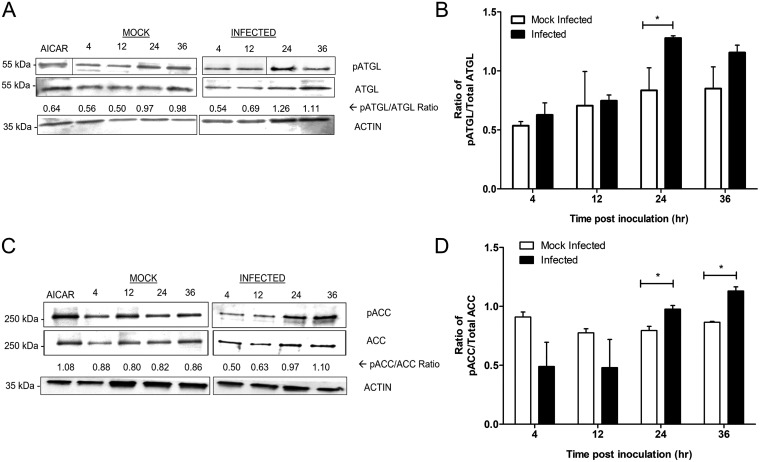
ATGL is activated, and ACC is inhibited, in F. tularensis-infected cells. (A) Representative Western blot of BMDM cell lysates infected with F. tularensis Schu S4 or mock infected. Samples were assessed for phospho-ACC (Ser79), total ACC, and actin (loading control). The relative ratio is the densitometry of phosphorylated over total protein after normalization to β-actin. (B) Quantitative analysis of the ratios of phospho-ACC (pACC) to total ACC compared to the ratios for mock-infected cells at the same time point. Asterisks represent significant differences between mock-infected and infected lysates as determined by Student’s unpaired *t* test. (C) Representative Western blot of BMDM cell lysates infected with F. tularensis Schu S4 or mock infected. Samples were assessed for phospho-ATGL (Ser406) and total ATGL. (D) Quantitative analysis of the ratios of phospho-ATGL to total ATGL compared to the ratios for mock-infected cells at the same time point. Bars represent the means ± SD for blots from 3 independent experiments. Asterisks represent significant differences between mock-infected and infected lysates as determined by Student’s unpaired *t* test. *, *P* < 0.05.

To further understand how F. tularensis exploits lipid droplet homeostasis, we investigated the regulation of host lipid metabolism during infection. Since inhibition of triacylglycerol degradation, specifically ATGL activity, negatively impacts F. tularensis replication in macrophages and MEFs, we reasoned that ATGL activity is altered during infection (12). ATGL is one of two enzymes involved in the breakdown of triacylglycerols within lipid droplets, which is a process known as lipolysis (25, 32). ATGL is the first and rate-limiting step of lipolysis and was shown to be essential for triacylglycerol catabolism (25, 33). One mechanism of ATGL activation is through its phosphorylation at serine residues. Recently, serine residue 406 has been characterized as being important for triacylglycerol hydrolase activity (26, 33). As triacylglycerols are degraded, fatty acids and glycerol molecules are freely available. To further facilitate the breakdown of fatty acids, the cell will undergo β-oxidation. Acetyl-CoA carboxylase (ACC), an enzyme that catalyzes the first step of *de novo* fatty acid biosynthesis, controls β-oxidation through the suppression of the fatty acid transport protein carnitine palmitoyl transferase 1 (CPT1) (Fig. 2A) (19, 20). However, the phosphorylation of ACC relieves the suppression of CPT1 and promotes the cell to undergo β-oxidation (19, 23, 32). Therefore, we assessed the phosphorylation of ATGL (Fig. 3A) and ACC (Fig. 3B) in mock- and F. tularensis Schu S4-infected BMDMs over a time course by Western blotting with phosphospecific antibodies. Compared to mock-infected BMDMs, the ratio of phospho-ATGL to total ATGL increased, with a significantly higher ratio of phospho-ATGL to total ATGL seen at 24 h p.i. (Fig. 3C). Furthermore, the ratio of phospho-ACC to total ACC increased over time in infected BMDMs. This ratio was statistically higher than that in mock-infected BMDMs at 24 and 36 h p.i. (Fig. 3D). These data suggest that lipolysis and β-oxidation are activated in F. tularensis-infected cells.

### AMPK supports F. tularensis intracellular growth.

**FIG 4:**
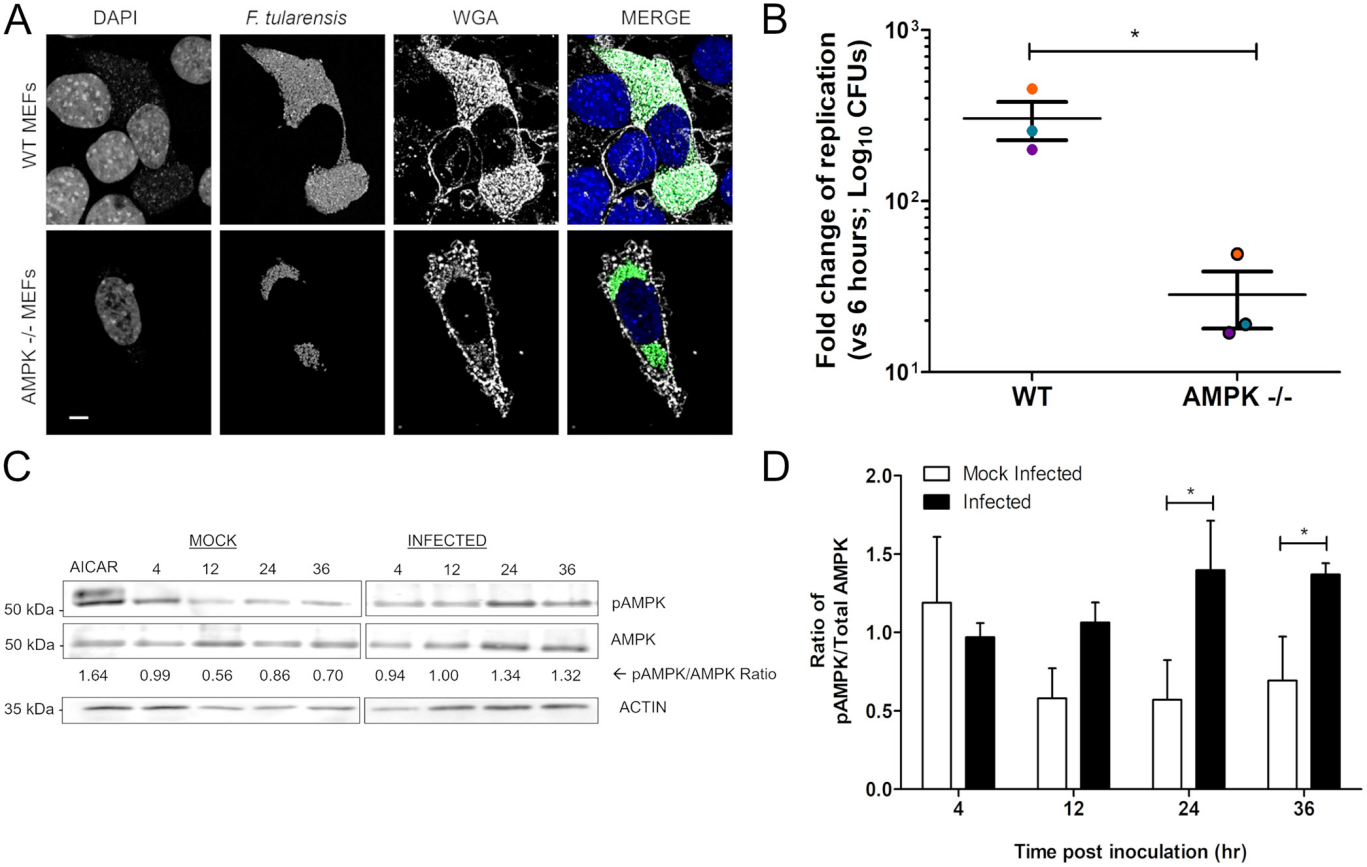
AMPK is activated during F. tularensis infection. (A) Representative confocal micrographs of WT and AMPK^−/−^ MEFs infected with WT F. tularensis Schu S4 expressing GFP for 36 h. Nuclei were stained using 4′,6-diamidino-2-phenylindole (DAPI). WGA (wheat germ agglutinin) was used to stain cell membranes. Bar, 5 μm. (B) WT and AMPK^−/−^ MEFs infected with WT F. tularensis Schu S4. Bacterial growth was measured via dilution plating (CFU per milliliter). The fold change in replication was calculated as the ratio of CFU at 36 h to CFU at 6 h. Data points are the means ± SD of results from 3 independent experiments performed in triplicate. Asterisks represent significant differences between changes in replication in WT MEFs and AMPK^−/−^ MEFs as determined by Student’s unpaired *t* test. *, *P* < 0.05. (C) Representative Western blot of BMDM cell lysates infected with F. tularensis Schu S4 or mock infected. Samples were assessed for phospho-AMPK (Thr172), total AMPK, and β-actin (loading control). The relative ratio is the densitometry of phosphorylated over total protein after normalization to β-actin. (D) Quantitative analysis of the ratios of phospho-AMPK to total AMPK compared to the ratios for mock-infected cells at the same time point after normalization to β-actin. Data represent the means ± SD for blots from 3 independent experiments. Differences between mock-infected and infected lysates were determined by Student’s unpaired *t* test. *, *P* < 0.05.

AMPK is a host protein that can activate or inhibit metabolic pathways, including lipolysis and lipid synthesis, to regulate cell metabolism (21). Many studies have shown that AMPK activity can be altered during infection (18, 19, 22). Additionally, AMPK can directly interact with ATGL and ACC to promote lipid metabolism (20, 25). We hypothesized that AMPK activation may contribute to nutrient availability in F. tularensis-infected cells. To test this hypothesis, we first assessed the bacterial replication of F. tularensis in the presence of an AMPK inhibitor, compound C, using a luminescence reporter assay (29, 34). The growth kinetics of virulent F. tularensis Schu S4 were significantly reduced with compound C (Fig. S2). Although compound C did not significantly affect bacterial growth in broth (Fig. S2), we observed a significant amount of cell death in a dosage-dependent manner (Fig. S2). These data suggest that the reduction of bacterial growth with compound C could be due to cell cytotoxicity during infection. Therefore, to conclude that AMPK activity supports F. tularensis replication, we decided to examine bacterial growth in AMPK knockout cells. AMPK knockouts are embryonic lethal, and monocyte knockout lines are unavailable. F. tularensis replicates in several different cell types, including MEFs, which are still relevant to infection. Additionally, an AMPK knockout cell line is available in MEFs. Therefore, we observed F. tularensis bacterial replication in AMPK-null (AMPK^−/−^) MEFs. WT and AMPK^−/−^ MEFs were infected with WT F. tularensis Schu S4 expressing green fluorescent protein (GFP) (Fig. 4A). We observed that WT MEFs at 36 h p.i. had many bacteria inside infected cells, whereas AMPK^−/−^ MEFs did not appear to sustain a high level of replication (Fig. 4A). Furthermore, we observed lower numbers of infected AMPK^−/−^ MEFs than infected WT MEFs (data not shown). To quantify these observations, WT and AMPK^−/−^ MEFs were infected with virulent WT F. tularensis Schu S4, and bacterial growth was measured at 6 h and 36 h p.i. via dilution plating. The change in growth between 6 h and 36 h p.i. quantified by dilution plating was 10-fold lower in AMPK^−/−^ MEFs than in WT MEFs (Fig. 4B), which suggests that AMPK^−/−^ MEFs do not support high levels of F. tularensis replication. To confirm that this reduction in bacterial replication was not due to delayed phagosome escape but was primarily due to a lack of nutrients, we infected WT and AMPK^−/−^ MEFs with WT F. tularensis Schu S4 and an F. tularensis type VI secretion system (T6SS) mutant (Δ*dotU*) and stained for late endosomal marker, lysosomal associated membrane protein 1 (LAMP-1) (10, 35). There was no significant difference in the proportions of WT bacteria associated with LAMP-1 in WT MEFs (25% of bacteria colocalized with LAMP-1) and AMPK^−/−^ MEFs (34% of bacteria colocalized with LAMP-1) (Fig. S3). As expected, the T6SS mutant had a significant delay in phagosome escape in both WT MEFs (62% of bacteria colocalized with LAMP-1) and AMPK^−/−^ MEFs (60% of bacteria colocalized with LAMP-1) (Fig. S2) (10). Altogether, these results support the conclusion that the observed growth defect in AMPK^−/−^ MEFs is independent of phagosome escape.

### AMPK is activated in F. tularensis-infected cells.

To better understand how AMPK activity supports F. tularensis replication, we examined the AMPK activation status in infected cells. AMPK is activated through phosphorylation at residue Thr172 in response to an increased AMP-to-ATP ratio, which is necessary for its catalytic activity (20). Therefore, we assessed the phosphorylation of AMPK in mock- and F. tularensis Schu S4-infected BMDMs over a time course by Western blotting using antibodies specific for phosphorylation at residue Thr172 (Fig. 4C) (19, 20). Compared to mock-infected BMDMs, the ratio of phospho-AMPK to total AMPK increased, with a significantly higher ratio at 24 h and 36 h p.i. (Fig. 4D). These data suggest that AMPK activation increases during infection. Altogether, these results support the conclusion that AMPK activation contributes to F. tularensis replication.

### AMPK activation is dependent on bacterial replication.

**FIG 5:**
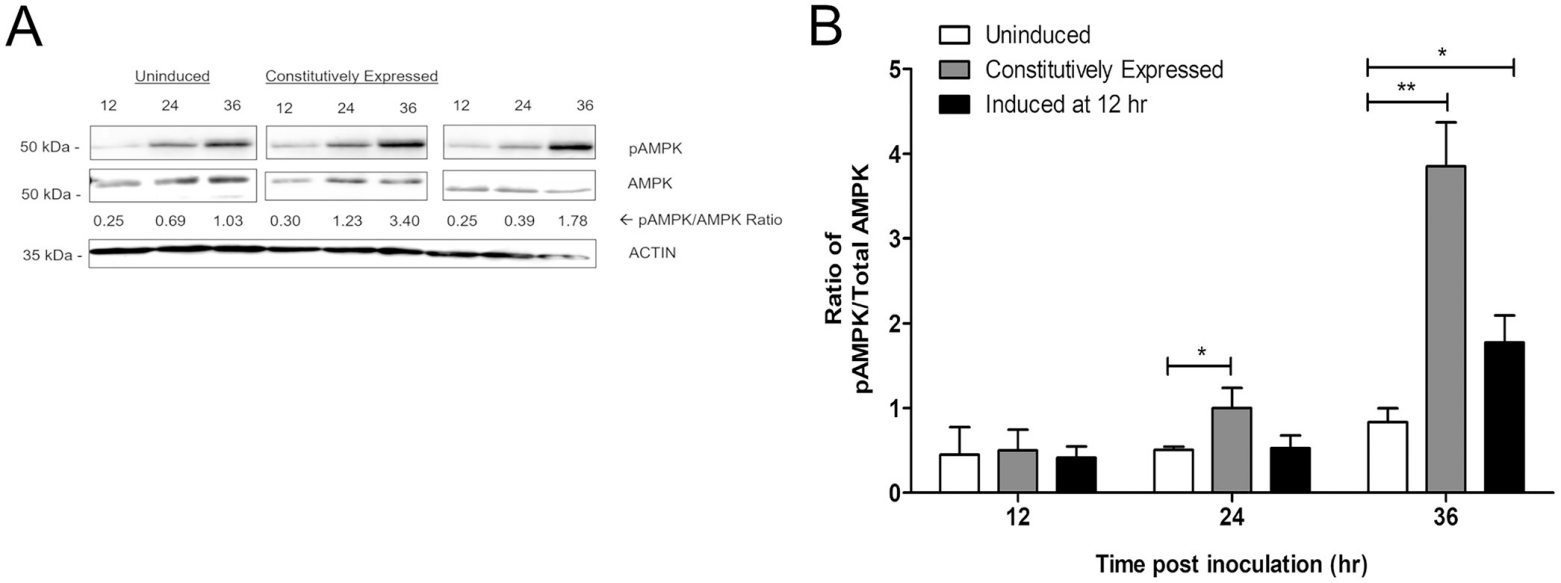
AMPK activation is dependent on bacterial replication. (A) Representative Western blot of BMDM cell lysates infected with F. tularensis Schu S4 Δ*ripA*::pEDL17*ripA*. Samples were assessed for phospho-AMPK (Thr172), AMPK, and β-actin (loading control). Uninduced, cells left untreated; Constitutively Expressed, cells in the continuous presence of ATc. The relative ratio represents the densitometry of phospho-AMPK to total AMPK. (B) Densitometry of phospho-AMPK and AMPK after normalization to β-actin. Bars represent the means ± SD for blots from three independent experiments. Induced at 12 hr, cells treated with ATc at 12 h p.i. Asterisks represent significant differences between the uninduced and test groups (i.e., either constitutively expressed or induced at 12 h) as determined by unpaired Student’s *t* test. *, *P* < 0.05.

We reasoned that AMPK could be activated directly through the activity of an F. tularensis effector or as a consequence of nutrient depletion caused by early bacterial replication and the theft of endogenous nutrients (36). To distinguish between these possibilities, we used an F. tularensis Schu S4 strain with tunable intracellular replication. Specifically, we used a *ripA* deletion mutant harboring a plasmid containing a tetracycline (TET)-inducible *ripA* gene (37). In the absence of the tetracycline analog anhydrotetracycline (ATc), this strain will escape the phagosome, enter the cytosol, and remain metabolically active, without measurable replication (37, 38). Once ATc is added (i.e., *ripA* expression is induced), this strain initiates replication and divides at the same rate as that of wild-type bacteria (Fig. S4) (37). We infected BMDMs with this strain and either left cells untreated or treated cells with ATc at 12 h p.i. As a control, we infected BMDMs with the same strain in the continuous presence of ATc to allow the constitutive expression of *ripA* and bacterial replication, which should resemble wild-type infection. Following lysis of cells, we probed for phospho-AMPK and total AMPK by Western blotting. As expected, the constitutive expression of *ripA* resulted in an increase in the ratio of phospho-AMPK to total AMPK, which was significantly higher at 24 and 36 h p.i. than in uninduced cells (Fig. 5). In the absence of intracellular replication (i.e., no ATc-induced expression of *ripA*), infected BMDMs had little change in the ratio of phospho-AMPK to total AMPK; however, we did see a slight increase in phospho-AMPK at 36 h p.i. (Fig. 5). With the addition of ATc at 12 h p.i., infected BMDMs showed an increase in the ratio of phospho-AMPK to total AMPK only after ATc was added, which was significantly higher at 36 h p.i. than in uninduced cells (Fig. 5B). Altogether, these data suggest that an increased bacterial burden leads to nutrient deprivation, resulting in the subsequent activation of AMPK.

## DISCUSSION

AMPK is a critical regulator of energy homeostasis in the cell, and following activation, it regulates many downstream metabolic pathways (20, 21). Some pathogens modulate AMPK activation to avoid host immune defense strategies such as autophagy or reactive oxygen species (ROS) production (39, 40). Other pathogens modulate AMPK activation to liberate host-derived nutrients as AMPK activation regulates fatty acid, lipid, and glucose metabolism (22, 39–41). AMPK regulates these pathways through direct interactions with downstream enzymes involved in both fatty acid synthesis and oxidation. Furthermore, AMPK regulates the expression of genes involved in glucose uptake and metabolism (42). In this study, we investigated the relationship between AMPK and F. tularensis infection and how host-derived nonglucose carbon sources become available in the intracellular environment of infected cells. We found that lipid droplets degrade during F. tularensis infection and that the regulation of AMPK and downstream lipid metabolism play important roles in F. tularensis infection. Altogether, our data suggest that F. tularensis exploits AMPK activation and AMPK-mediated lipid metabolism to harvest nutrients sequestered in lipid droplets.

Our laboratory and others have demonstrated that the availability of host-derived nutrients impacts F. tularensis intracellular replication (11, 12, 14). For example, F. tularensis is auxotrophic for 13 amino acids and relies exclusively on amino acid uptake systems (11, 13, 43). F. tularensis harvests host-derived amino acids for protein synthesis and energy to undergo intracellular replication (11). In addition to amino acids, F. tularensis depends on gluconeogenic carbon sources such as glycerol, rather than glycolytic sources, as energy to support replication (12). Lipid droplets, in which glycerol is sequestered, are a major nutrient source for bacterial pathogens. For Mycobacterium tuberculosis and Chlamydia trachomatis, the accumulation of lipid droplets is essential to harvest triglycerides and fatty acids (44–46). For other bacteria such as Coxiella burnetii, host lipolysis contributes to replication and provides nutrients necessary for replication (30). In this study, we found that infected cells had significantly smaller and fewer lipid droplets than uninfected cells, which highlights the importance of lipid droplet degradation for F. tularensis (Fig. 1). We also found that altering lipid droplet homeostasis resulted in various effects on F. tularensis intracellular growth (Fig. 2). Similar results were seen with Coxiella burnetii, in which bacterial replication kinetics were assessed when lipid droplet formation and degradation were inhibited (30). For C. burnetii, replication appeared to increase with the inhibition of lipid droplet formation, while replication decreased when lipid droplet degradation was inhibited (30). In this study, we found that the inhibition of the formation of triacylglycerols, which make up a large, a large portion of the neutral core of lipid droplets, had no impact on F. tularensis replication in MEFs and primary macrophages; however, the inhibition of triacylglycerol degradation significantly reduced bacterial growth in both cell types (Fig. 2). These results suggest that F. tularensis uses nutrients sequestered in lipid droplets and that the inhibition of lipid droplet formation and/or the activation of lipid droplet degradation allows F. tularensis access to crucial nutrients sequestered in lipid droplets (27, 32, 47).

Lipid droplets can be degraded by two distinct mechanisms, lipolysis and lipophagy (27, 48). Lipolysis depends on ATGL activity, whereas lipophagy is dependent on autophagic machinery (27, 48). We observed that F. tularensis infection induced lipolysis by upregulating the phosphorylation of ATGL (Fig. 3). We speculate that lipophagy is dispensable as F. tularensis infection induces noncanonical autophagy independent of components such as Atg5 and mTOR (11, 27, 48). We also observed an increase in the ratio of phospho-ACC to total ACC in infected cells over time (Fig. 3). The phosphorylation of ACC downregulates lipid synthesis, which causes a subsequent increase in lipid breakdown and the trafficking of fatty acids to the mitochondria (24–26). We therefore conclude that the induction of lipolysis, through the activation of ATGL and inhibition of ACC, allows F. tularensis to harvest nutrients from lipid droplets.

Evading pathogens can reprogram host metabolism and cause metabolic stress to favor replication and survival (49). In this study, we found that inhibition of AMPK activation with compound C reduced bacterial loads in infected cells by 10-fold; however, there were indirect cytotoxic effects on host cells (Fig. S2). Similar results have been seen in compound C-treated cancer cell lines as treatment halted cell proliferation independent of AMPK (50). We could not discount the possibility that the induction of cell death by compound C during infection negatively impacted F. tularensis intracellular growth (50). Therefore, we decided to use AMPK^−/−^ MEFs to accurately identify the relationship between AMPK activity and bacterial replication. We found that in AMPK^−/−^ MEFs, F. tularensis replication decreased by 10-fold (Fig. 4). More importantly, we found that AMPK^−/−^ MEFs did not delay the phagosome escape kinetics of F. tularensis (Fig. S3), which suggests that the reduction in replication kinetics is due to limited host-derived nutrients. However, F. tularensis bacterial replication was not completely abrogated, as there was still some level of replication observed in AMPK^−/−^ MEFs (Fig. 3A), suggesting that F. tularensis may replicate slower in AMPK^−/−^ MEFs. Altogether, we conclude that the activation of AMPK is not the sole mechanism behind nutrient liberation, but it does provide evidence that the regulation of AMPK activity can impact F. tularensis replication.

As pathogens replicate, nutrients are quickly exhausted, causing cellular starvation, which can trigger the cell to reprogram its cellular metabolism (2, 4). We found that at 24 h p.i., the ratio of phospho-AMPK to total AMPK increased compared to that in mock-infected cells. When we infected BMDMs with a tunable deletion mutant to control bacterial replication and determine if AMPK activation was dependent on the active replication of F. tularensis, we found that in the absence of intracellular replication, infected BMDMs had little change in the ratio of phospho-AMPK to total AMPK (Fig. 5). However, we did see a slight increase in phospho-AMPK at 36 h (Fig. 5). We speculate that this increase in the activation of AMPK may be due to the strain still being metabolically active. Although there is no expression of *ripA* and, therefore, the bacteria do not undergo replication, the *ripA* mutant is still viable in the host cell and may alter the metabolic state of cells during infection. Despite this, we observed significantly higher activation of AMPK when *ripA* expression was induced and bacterial replication was induced (Fig. 5). Altogether, these data suggest that AMPK activation increases in response to an increased bacterial burden. We conclude that AMPK activation is due to metabolic stress (i.e., nutrients decrease when bacterial replication increases), which leads to the AMPK-mediated phosphorylation of ATGL and ACC to increase lipolysis (20, 24, 25). As a result, F. tularensis exploits this process to harvest host-derived nutrients sequestered in lipid droplets and undergo efficient intracellular replication.

**FIG 6:**
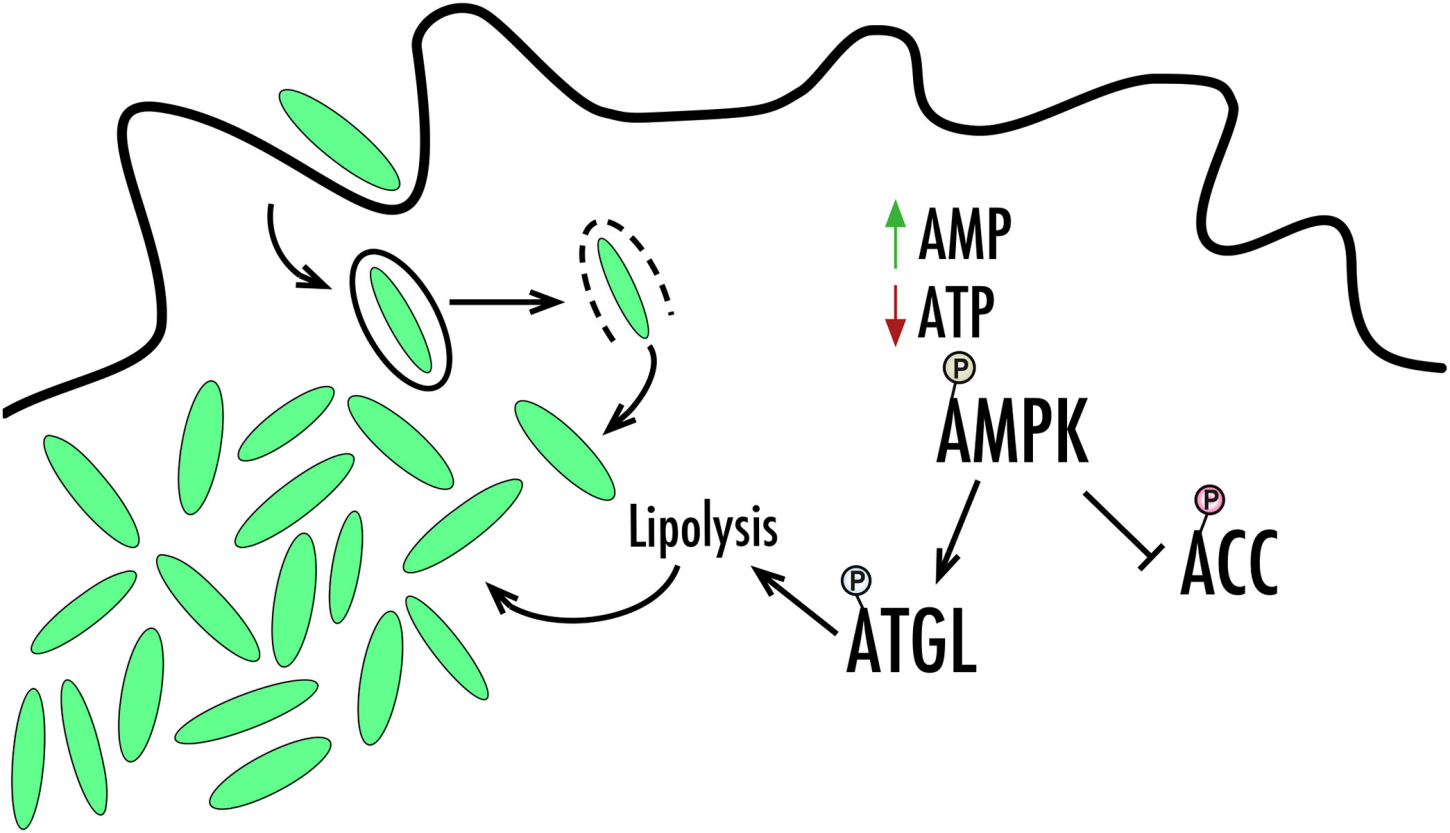
Model of modulation of AMPK activation and downstream lipid metabolism. As bacterial replication occurs in the cytoplasm, nutrients begin to be depleted, and the host cell activates AMPK to promote the degradation of nonessential nutrient stores. Increased lipid droplet breakdown mediated by ATGL through lipolysis liberates important nutrients that support intracellular bacterial growth. Additionally, inhibition of ACC through phosphorylation pushes the host cell to undergo fatty acid oxidation, which can also promote intracellular bacterial growth.

In this study, we demonstrate that AMPK activation allows F. tularensis to harvest important nutrients (summarized in Fig. 6). However, future studies are needed to investigate if AMPK also contributes to the regulation of immune responses during F. tularensis infection. AMPK activation, in response to metabolic stress, can regulate effective immune cell responses (51). In fact, AMPK activation has been shown to be involved in the activation of antimicrobial immunity during mycobacterial infection (52). It is possible that targeting AMPK activation or modulating the kinetics in which it occurs during F. tularensis infection could be a promising strategy to combat this bacterium. While we demonstrated that fatty acid oxidation is upregulated during infection, it is unclear if F. tularensis can alter the trafficking of fatty acids and use these nutrients as energy for replication. Therefore, further studies are also needed to determine the importance of fatty acids during F. tularensis infection. Overall, our findings highlight a new host-pathogen interaction for F. tularensis to exploit the cell’s nutrients, which provides insight into F. tularensis pathogenesis.

## MATERIALS AND METHODS

### Bacterial strains.

The Francisella tularensis subsp. *holarctica* live vaccine strain (LVS) was obtained from the Centers for Disease Control and Prevention (Atlanta, GA), and F. tularensis subsp. *tularensis* Schu S4 was obtained from BEI Resources. Wild-type (WT) F. tularensis Schu S4 was initially cultured on chocolate agar supplemented with 1% IsoVitaleX (see the supplemental material for the recipe). WT F. tularensis Schu S4 harboring a luciferase (LUX) plasmid (pJB3 [29]) and WT F. tularensis LVS pEDL20 (53) were cultured on chocolate agar supplemented with 1% IsoVitaleX and 200 μg/mL hygromycin (Cayman Chemicals). WT F. tularensis Schu S4 expressing green fluorescent protein (GFP) was cultured on chocolate agar supplemented with 1% IsoVitaleX and 10 μg/mL kanamycin (Sigma-Aldrich). All strains were grown overnight at 37°C with aeration in Chamberlain’s defined broth medium (CDM) following growth on plates (54).

### Cell culture.

Bone marrow-derived macrophages (BMDMs) were generated from 6- to 10-week-old female C57BL/6 mice (Jackson Laboratories) as previously described (55). Cells recovered from murine femurs were incubated for 5 days in Dulbecco’s modified Eagle medium (DMEM) with 4.5 g/L glucose (Corning) containing 10% heat-inactivated fetal bovine serum (FBS) (R&D Systems), 20 ng/mL recombinant mouse macrophage colony-stimulating factor (MCSF) (Peprotech), 1 mM l-glutamine (Gibco), 1 mM HEPES (Gibco), and 1 mM nonessential amino acids (Cytiva) (complete DMEM [cDMEM]). On day 2, nonadherent cells were spun down and reseeded in new cDMEM. On day 4, cells were refed with fresh cDMEM and seeded on day 5 for infections. BMDMs were routinely checked for macrophage markers, with >90% of cells being positive for F4/80 and CD11b and negative for CD11c (data not shown). Mouse embryonic fibroblasts (MEFs) were maintained in DMEM with 4.5 g/L glucose and 10% heat-inactivated FBS. Wild-type and AMPK-deficient (AMPK^−/−^) MEFs were a generous gift from Nathanial Moorman from the University of North Carolina—Chapel Hill, Chapel Hill, NC. J774A.1 (ATCC TIB-67) macrophages were maintained in DMEM supplemented with 4.5 g/L glucose, 10% heat-inactivated FBS, 2 mM l-glutamine, and 1 mM sodium pyruvate (Cytiva). All tissue culture lines were maintained at 37°C with 5% CO_2_.

### Drug treatments.

Compound C (Cayman Chemicals) was suspended in dimethyl sulfoxide (DMSO) at a stock concentration of 10 μM and added to J774A.1 macrophages at the indicated concentrations 1 h prior to bacterial inoculation. The compound C dosage was determined based on data from previous studies (50). Compound C remained on macrophages throughout infection. The cytotoxicity of compound C in J774A.1 macrophages was determined using a Vybrant 3-(4,5-dimethyl-2-thiazolyl)-2,5-diphenyl-2H-tetrazolium bromide (MTT) cell proliferation assay kit (Thermo Fisher) according to the manufacturer’s protocol. Atglistatin (30 μM) (Cayman Chemicals), triacsin C (10 μM) (Enzo Life Sciences), and T863 (10 μM) (Cayman Chemicals) were suspended in DMSO (12, 30). For lipid inhibitor studies, we pretreated cells overnight with compounds and continued treatment throughout infection.

### Growth curves.

Bacterial growth curves were performed by measuring the optical density at 600 nm (OD_600_) (every 15 min) using an Infinite M200 Pro plate reader (Tecan), maintaining a constant temperature (37°C). For all growth kinetics, bacteria were grown overnight in CDM and then diluted to an OD_600_ of 0.10 to 0.15 in fresh CDM. For the assessment of the direct effects of compound C and atglistatin, bacterial cultures were diluted in CDM with the indicated concentrations of compound C or atglistatin.

### Intracellular infections.

Bacterial intracellular growth kinetics were determined by measuring the luminescence of F. tularensis Schu S4 LUX-infected cells, as previously described (29). Cells were seeded at a density of 5 × 10^4^ cells/well in 96-well white-wall white-bottom tissue culture-treated plates (Corning). Cells were inoculated at a multiplicity of infection (MOI) of 100. The medium was removed and replaced with medium containing 25 μg/mL of gentamicin (Gibco) at 2 h p.i. (J774A.1 macrophages or BMDMs) or 3 h p.i. (MEFs). Luminescence was measured every 15 min using an Infinite M200 Pro plate reader (Tecan), maintaining constant temperature (37°C) and carbon dioxide (5%). To enumerate intracellular bacteria by calculating the CFU per milliliter, cells were plated at 1 × 10^5^ cells/well into a 24-well tissue culture-treated plate (Corning). Cells were inoculated with F. tularensis Schu S4 at an MOI of 100. The medium was removed and replaced with medium containing 25 μg/mL of gentamicin at 2 h p.i. (J774A.1 macrophages or BMDMs) or 3 h p.i. (MEFs). At the indicated time points, cells were washed once with phosphate-buffered saline (PBS) before being scraped, vortexed for 1 min, diluted, and plated onto chocolate agar.

### Immunofluorescence.

MEFs were seeded at a density of 5 × 10^4^ cells/well in a 12-well tissue culture-treated plate containing 8-mm round glass coverslips (Matsunami). Cells were inoculated with F. tularensis Schu S4 or F. tularensis Schu S4 GFP at an MOI of 100. Medium was replaced at 3 h p.i. with medium containing 25 μg/mL of gentamicin. For wheat germ agglutinin (WGA) staining, cells were fixed for 15 min with 4% (wt/vol) paraformaldehyde at 36 h p.i. After fixation, cells were permeabilized with 0.1% saponin in PBS with 2% FBS (block buffer) for 45 min. Cells were washed and stained with WGA-tetramethylrhodamine isothiocyanate (TRITC) (Thermo Fisher) (1:250) for 7 min at room temperature (RT). Cells were analyzed by confocal fluorescence microscopy (SP8 point scanning confocal microscope; Leica) to identify infected cells. For LAMP-1 colocalization, cells were fixed for 15 min with 4% (wt/vol) paraformaldehyde at 6 h p.i. After fixation, cells were permeabilized with 0.1% saponin in PBS with 2% FBS (block buffer) for 45 min. Cells were washed and stained with a rat anti-LAMP-1 antibody (1:500) (catalog number 1D4B-c; Developmental Studies Hybridoma Bank [DSHB]) and goat anti-rat IgG–TRITC secondary antibody (catalog number 26-4826-82; Invitrogen). Cells were analyzed by confocal fluorescence microscopy (SP8 point scanning confocal microscope; Leica) to identify infected cells. Images were analyzed using Leica microscopy software. Blinded images were quantified for the association of bacteria with LAMP-1 using ImageJ. For lipid droplet quantification, cells were fixed for 15 min with 4% (wt/vol) paraformaldehyde at 36 h p.i. and stained with BODIPY 493/503 (1:500) (catalog number D3922; Invitrogen) and an anti-F. tularensis lipopolysaccharide (LPS) (catalog number F6070-02X; U.S. Biologicals)-conjugated Alexa Fluor 647 antibody (1:250) made in-house using an Alexa Fluor 647 labeling kit (Thermo Fisher). Cells were washed and analyzed by confocal fluorescence microscopy (SP8 point scanning confocal microscope; Leica) for F. tularensis infection. Images of highly infected cells (>10 bacteria) were taken. All images were analyzed in a blind manner for droplet number, droplet area, and droplet mean fluorescence intensity (MFI) using ImageJ particle analysis. For lipid droplet quantification, triacsin C- and T863-treated cells were fixed at 16 h posttreatment and stained as described above.

### Western blotting.

For Western blotting, BMDMs were seeded at a density of 5 × 10^5^ cells/well in 6-well clear-bottom tissue culture-treated plates (Corning). Cells were inoculated with WT F. tularensis Schu S4 at an MOI of 100 and treated with gentamicin at 2 h p.i. Mock-infected samples had medium replaced at 2 h p.i. with medium containing gentamicin. AICAR (200 μm) (Cayman Chemicals) was added and remained on the cells for 1 h. At the indicated times, cells were lysed with 4× SDS lysis buffer with 1× Halt protease and phosphatase inhibitor cocktail (Thermo Scientific) and 1% β-mercaptoethanol (BME). Lysates were separated on SDS-PAGE gels under reducing conditions and transferred to a polyvinylidene difluoride (PDVF) membrane. Membranes were blocked with 3% milk in Tris-buffered saline–Tween (TBST) for 1 h and then probed with primary antibodies to AMPK (1:1,000) (catalog number 2532; Cell Signaling), phospho-AMPK (Thr172) (1:1,000) (catalog number 2535; Cell Signaling), ATGL (1:1,000) (catalog number 2138; Cell Signaling), phospho-ATGL (Ser406) (1:2,000) (catalog number AB135093; Abcam), ACC (1:1,000) (catalog number 3662; Cell Signaling), phospho-ACC (Ser79) (1:1,000) (catalog number 3661; Cell Signaling), and mouse anti-β-actin (1:5,000) (catalog number MAB8929; Fisher) overnight in 5% bovine serum albumin (BSA) in TBST. Membranes were then probed with horseradish peroxidase-conjugated anti-rabbit IgG (1:5,000) (catalog number 7074; Cell Signaling) or horseradish peroxidase-conjugated anti-mouse IgG (1:5,000) (catalog number 7076; Cell Signaling). Bands were developed using the SuperSignal West Femto maximum-sensitivity substrate (Thermo Scientific).

Densitometry analysis was performed using ImageJ’s gel analysis function. Each band density was measured, and densities are expressed as the ratio of phosphorylated protein to total protein.
