# Supplementary material for: Francisella tularensis Exploits AMPK Activation to Harvest Host-Derived Nutrients Liberated from Host Lipolysis
Source: Infect Immun. 2022 Aug 2;90(8):e00155-22. doi: 10.1128/iai.00155-22 (PMC9387300; doi:10.1128/iai.00155-22)
Supplement: Supplemental file 1 — Supplemental material. Download iai.00155-22-s0001.pdf, PDF file, 0.5 MB [file iai.00155-22-s0001.pdf]

Supplemental materials and methods

Isovitalex Recipe

**Chemical**	**Stock concentration**	**Solvent**	**Volume needed for 1 L**
Dextrose	100 g/250 ml	Water	250 ml
Guanine HCl	30 mg/10 ml	0.1N NaOH	10 ml
L-glutamine	10g/250ml	Warm water	250 ml
Thiamine HCl	9mg/30ml	Water	10 ml
Adenine	1g/50ml	0.2N HCl	50 ml
NAD	250mg/10ml	Water	10 ml
Vitamin B12	10mg/10ml	Water	10 ml
4-aminobenzoic acid (PABA)	13mg/10ml	Water	10 ml
Thiamine pyrophosphate	100mg/10ml	Water	10 ml
Ferric nitrate	20mg/10ml	Water	10 ml
L-cystine	1.1g/100ml	0.5N HCl	100 ml
L-cysteine HCl	25.9g/100ml	Water	100 ml

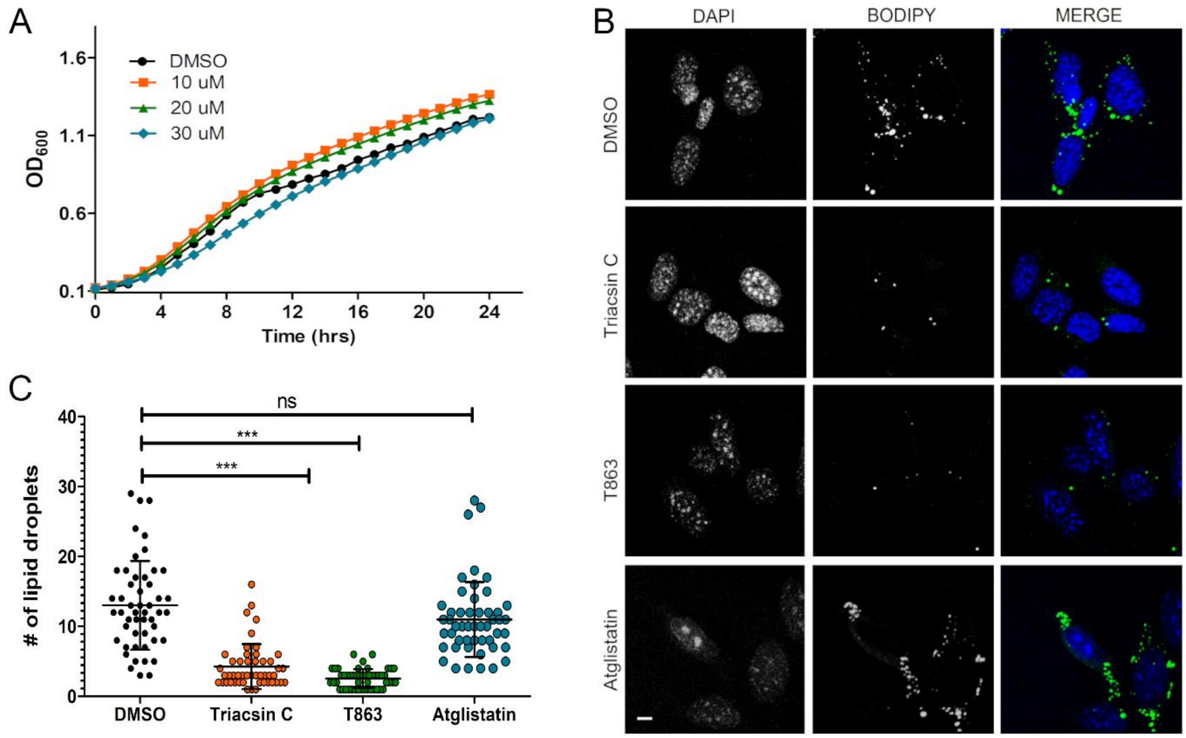

Figure S1: (A) Growth kinetics of WT *F. tularensis* Schu S4 in CDM with DMSO or various doses of atglistatin. Data points are mean of 3 independent experiments performed in triplicate. **(B)** Representative confocal micrographs of WT MEFs treated overnight with 5 µM triacsin C, 10 µM T863, or 30 uM atglistatin. Cells were stained with BODIPY 493/503. Scale bar represents 5 µM. **(C)** Quantification of number of lipid droplets per cell (N=50 cells). Asterisk represents the significant difference as determined by one-way ANOVA and Dunnett's post-hoc test. *** p<0.001.

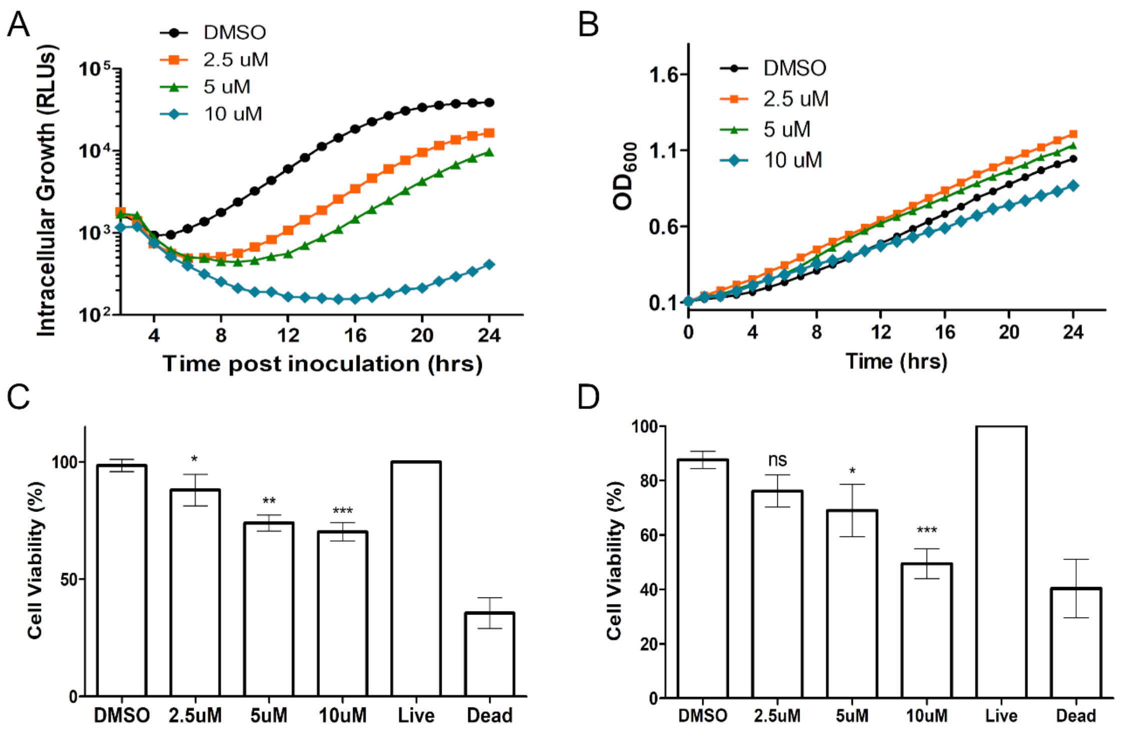

Figure S2: (A) Growth kinetics of WT *F. tularensis* Schu S4 harboring a luciferase plasmid (LUX) in J774A.1 macrophages. Luminescence (RLUs) was measured over 24 hrs. Data points are the mean from 3 independent biological replicates performed in triplicate. Asterisks represent significant difference between DMSO treated cells and 5 µM Compound C treated cells (blue) or 10 µM Compound C treated cells (purple) determined by one-way ANOVA and Dunnett's post-hoc test. * $p<0.05$, ** $p=0.004$, *** $p=0.002$ **(B)** Growth kinetics of WT *F. tularensis* Schu S4 in CDM with DMSO or various doses of Compound C. Data points are mean of 3 independent experiments performed in triplicate. **(C)** Cell viability of *F. tularensis* infected J774A.1 with various doses of Compound C at 8 hrs. Data points are mean ± SD of 3 independent experiments performed in triplicate. Asterisks represent significant difference between DMSO control and Compound C treated cells determined by one-way ANOVA and Dunnett's post-hoc test. **(D)** Cell viability of *F. tularensis* infected J774A.1 with various doses of Compound

C after 16 hrs of treatment. Data points are mean ± SD of 3 independent experiments performed in triplicate. Asterisks represent significant difference between DMSO control and Compound C treated cells determined by one-way ANOVA and Dunnett's post-hoc test. Live and dead represent untreated cells. * $p < 0.05$

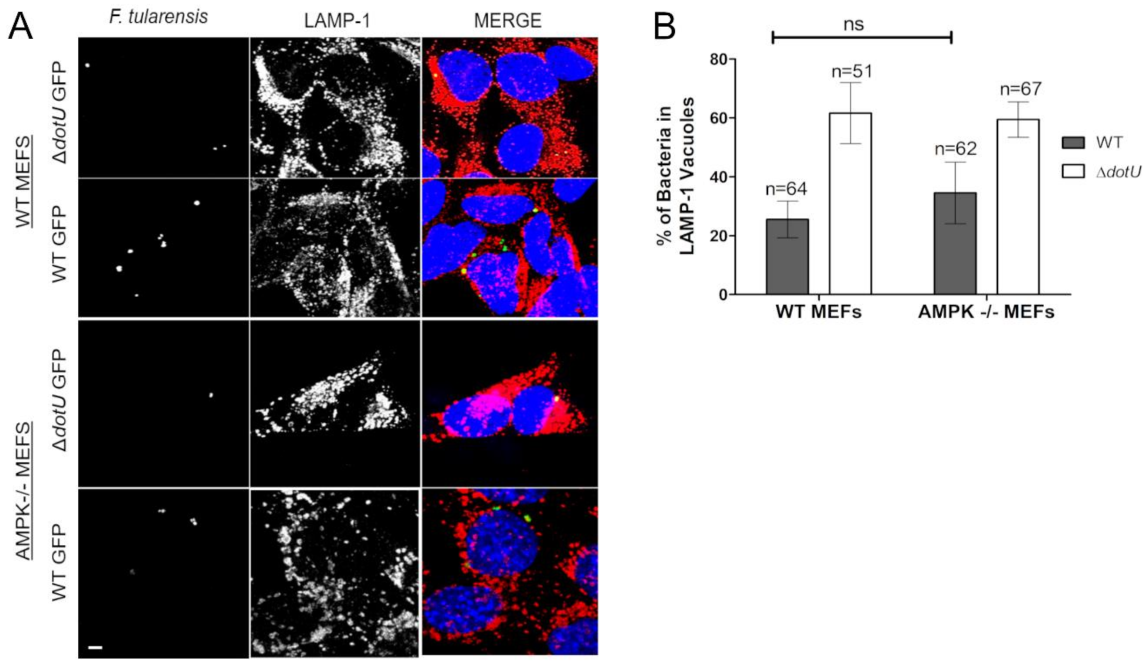

Figure S3: (A) Representative confocal fluorescence micrographs of WT and AMPK -/- MEFs infected with GFP expressing WT *F. tularensis* Schu S4 or GFP expressing Δ*dotU* Schu S4 for 6 hrs pi. Nuclei were stained using DAPI. Scale bar represents 5 µm. **(B)** WT and AMPK -/- MEFs were infected as in (A)**.** At 6hrs pi cells were fixed and immunostained for lysosomal marker LAMP-1. The number of bacteria associated with LAMP-1 staining was quantified by fluorescence microscopy. Data represents the mean ± SD of >3 independent experiments. N refers to the number of cells analyzed.

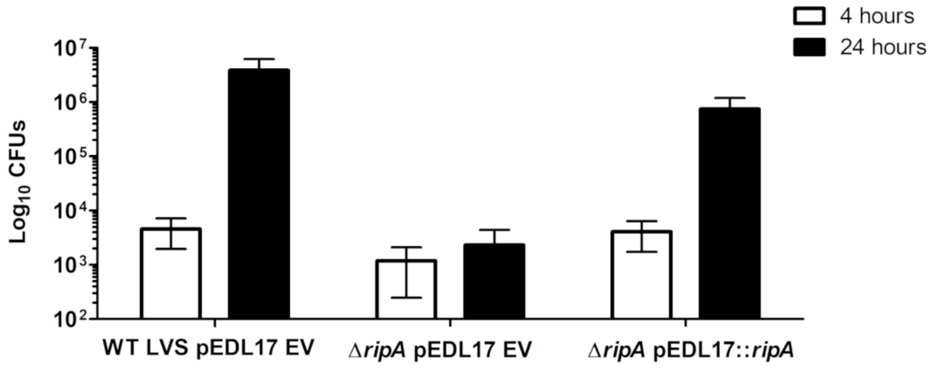

Figure S4: BMDMs infected with WT *F. tularensis* LVS S4 pEDL17 empty vector (EV), Δ*ripA* pEDL17 EV, and Δ*ripA*::pEDL17*ripA*. Bacterial growth was measured via dilution plating at 4 hours and 24 hours pi. Cells were treated with 100 µg/ml of ATc at 12 hours pi to confirm plasmid and rescue growth of Δ*ripA*::pEDL17*ripA*. Data points are the mean ± SD of 3 independent experiments performed in triplicate.
